# Personalized diabetes management: what do patients with diabetes mellitus prefer? A discrete choice experiment

**DOI:** 10.1007/s10198-021-01264-6

**Published:** 2021-02-15

**Authors:** Axel C. Mühlbacher, Andrew Sadler, Christin Juhnke

**Affiliations:** 1 Gesundheitsökonomie und Medizinmanagement, Hochschule Neubrandenburg, Brodaer Straße 2, 17033 Neubrandenburg, Germany; 2 Gesellschaft Für Empirische Beratung GmbH (GEB), Freiburg, Germany; 3 Department of Population Health Sciences, Duke University, Durham, NC USA

**Keywords:** Type II diabetes mellitus, Personalized diabetes management, Discrete choice experiment, Latent class analysis, I12 Health Behavior, C35 Discrete Regression and Qualitative Choice Models, Discrete Regressors, Proportions, C44 Operations Research, Statistical Decision Theory

## Abstract

**Background:**

There are unresolved procedural and medical problems in the care of diabetes, which cause high costs for health systems. These include the inadequate glycemic adjustment, care gaps, therapeutic inertia, and a lack of motivation. Personalized diabetes management can be seen as a kind of “standard process” that provides both physicians and patients with a framework. The aim of this empirical survey is the evaluation of patient preferences regarding personalized diabetes management. The purpose of this experiment is to demonstrate the properties of the programs that are relevant for the choice of insulin-based therapy regimens for patients with type II diabetes mellitus.

**Methods:**

A discrete choice experiment (DCE) was applied to identify preferences for a personalized diabetes management in patients with type II diabetes mellitus. Six attributes were included. The DCE was conducted in June 2017 using a fractional factorial design, and the statistical data analysis used random effect logit models.

**Results:**

*N* = 227 patients (66.1% male) were included. The preference analysis showed dominance for the attribute “occurrence of severe hypoglycemias per year” [level difference (LD) 2765]. Preference analysis also showed that participants weight the “risk of myocardial infarction (over 10 years)” (LD 1.854) highest among the side effects. Within the effectiveness criterion of “change in the long-term blood glucose level (HbA1c)” a change at an initial value of 9.5% (LD 1.146) is weighted slightly higher than changes at 7.5% (LD 1.141). Within the random parameter logit estimation, all coefficients proved to be significantly different from zero at the level *p* ≤ 0.01. The latent class analysis shows three heterogeneous classes, each showing clearly different weights of the therapeutic properties. This results in a clear three-folding: for 1/3 of the respondents the change of the long-term blood sugar (HbA1c value) is the top objective. Another third is solely interested in the short-term effectiveness of the therapy in the sense of the occurrence of severe hypoglycemias per year. The last third of the interviewees finally focuses on the follow-up regarding cardiovascular events. Overall, there were five structural and personality traits which have an influence on the respective probability of the class membership.

**Discussion/conclusion:**

This study identifies and weights the key decision-making criteria for optimal management of diabetes from the perspective of patients. It was shown that the effectiveness of a care program is the most important from the perspective of the patient and avoiding severe a hypoglycemia has the greatest influence on the choice. The risk of myocardial infarction as a follow-up disease and the long-term adjustment of the blood glucose follow the importance. In the analysis of possible subgroup differences by means of latent class analysis, it was found that three preference patterns exist within the sample. The generated preference data can be used for the design of personalized management approaches. It remains open to the extent to which expert opinions and patient preferences diverge.

## Decision-making context: diabetes mellitus type II and personalized diabetes management

### Diabetes mellitus

Diabetes mellitus is one of the most elaborate and expensive widespread diseases in Germany [1]. The high socioeconomic importance of diabetes is the result of severe complications, reduced life expectancy and reduced ability to work as well as the necessary comprehensive medical care for the patients [2].

It is estimated that about 8–10 million people are currently suffering from this incurable disease in the Federal Republic of Germany [3]. It is estimated that about twice as many people will be affected by diabetes by 2020 [4, 5]. The International Diabetes Federation estimates that by 2030 the worldwide number of people with diabetes will have increased from currently 285 million by more than 50% to about 438 million [6].

Diabetes mellitus is a glucose metabolism disorder that is characterized by chronic hyperglycemia [7]. A variable combination of impaired insulin secretion and/or reduced insulin resistance results in type II diabetes [8, 9]. This is the most common form of diabetes, which is diagnosed in about 90% of diabetic patients and is mostly only manifested in adulthood. This type of diabetes is characterized by a decreased insulin effect and a reduction in the insulin distribution and occurs more and more frequently also in younger people. Reasons for this are factors such as overweight, lack of exercise and incorrect diet, which significantly increases the risk of developing diabetes.

Studies have shown that diabetics have a lower quality of life than non-diabetics—especially patients with diabetes-related complications, as there is a risk of blindness, dialysis, or amputation of limbs. Likewise, the disease often causes emotional stresses—mental disorders such as depression occur twice as frequently in diabetics than in the normal population [8, 10, 11]. Hence, co-morbidities and secondary diseases of diabetes can lead to a reduction in the quality of life and a shortened life.

### Unresolved problems

Diabetes mellitus is a major challenge for national health systems, both regarding the care processes and the associated expenditures. The changed lifestyle and demographic, social changes result in a high burden due to type II diabetes [12]. The care of patients with type II diabetes is performed on three levels: (1) about 80–90% are under an outpatient care, (2) 10–20% of patients are either permanently or temporarily dependent on an outpatient care unit, a diabetic specialist or a hospital outpatient clinic, as well as coordinating specialists (e.g. nephrologists, ophthalmologists, etc.)—especially when acute and/or complications occur. An escalation of the treatment of acute and/or long-term complications necessitates part-time inpatient care (3) [13].

There are unresolved procedural and medical problem in the care of diabetes, which cause high costs for the health systems. These include in particular inadequate glycemic attitude, care gaps, therapeutic inertia as well as lack of motivation. The latter leads to a lack of therapy compliance and adherence of the patients and possible misunderstandings in the physician–patient communication [14].

The achievement of better therapeutic outcomes or the utilization of the therapeutic potential is often prevented by therapeutic inertia. Today, there are a variety of possibilities in differential therapy. However, these are not uniformly presented in recommendations for action and care guidelines for type II diabetes [15]. This often results in lengthy decision-making processes. The consecutive and consistent implementation of the therapeutic recommendations will be delayed. As a result, standards and an integrated care programs are required in diabetes care [15].

### Personalized diabetes management

Despite all efforts to achieve the best possible diabetic therapy in Germany, an insufficient glycemic adjustment (HbA1c level ≥ 7.5%) is found in almost half of the patients in type II diabetes [12]. A poor glycemic adjustment promotes the morbidity of patients with type II diabetes, especially the risk of developing micro- and macrovascular sequelae [16, 17].

Patients demand an individual/individualized therapy, even if the necessary motivation for (lasting) change in their lifestyles is missing at the same time. This not at least because doctors, as companions of the patients, can spend too little time and have little information about the patient’s preferences [18].

Diabetes is a chronic disease that places high demands on the compliance and adherence of patients. The success of the therapy is determined by various factors such as compliance with the recommended blood glucose monitoring or the documentation of blood glucose profiles. The use of a personalized approach to diabetes management is discussed internationally and the success has been demonstrated in first studies. Studies show that Personalized Diabetes Management (PDM) can increase the effectiveness and efficacy of therapy and improve treatment outcomes [19–22].

The PDM can be understood as a kind of “standard process” that provides a framework for both physicians and patients. It stands for a personalized diabetes care tailored to the specific patient. Structured, therapy-adapted blood glucose self-monitoring is linked to electronic systematic analysis and graphical presentation, interpretation and communication of the patient’s blood glucose measurements and diabetes data using a diabetes management software solution. This integrated therapy process is intended to enable improved quality of life, optimized therapy or optimized therapeutic outcomes (medical benefits) and economic added value. Personalized diabetes management thus has the task to realize an optimized diabetes care. Ultimately, the question of which tools and functionalities are used also depends on the needs and preferences expressed by the affected patients.

## Method, study design and decision-making model: how can preferences be measured?

### Method of the discrete choice experiment (DCE)

The discrete choice experiment (DCE) is a choice-based variant of the conjoint analysis, which became possible only through the theoretical work of Lancaster [23] and McFadden [24]. Instead of a ranking or evaluation of many different therapeutic features, a comparison of alternatives is made and a (choice) decision is made between different therapy options [25]. By means of the paired comparison, the degree of complexity of the task drops considerably for the patients, so that better data quality is to be expected [26–28]. The implementation of the discrete choice analysis also offers practical advantages due to its close proximity to reality. Hence, it has already emerged to one of the most frequently used preference measurement methods in health economic evaluation [29].

The structure of a discrete choice experiment and its analysis are multi-stage [26, 30–32]. The utility function is estimated by means of the maximum likelihood method. Depending on the underlying distribution function, different estimation methods (mostly probit or logit estimates) can be used [26, 29, 33–35].

### Literature review and AHP

In a first step, a literature review (PubMed, Medline and Cochrane Library) was conducted on clinical aspects of insulin therapy in diabetes mellitus type II and its management. The aim of the search was to identify the potential properties and characteristics of these treatments, in general and from the patients’ perspective. Based on the found full texts, a list of therapeutic and management features was created, discussed with experts and patient-relevant characteristics were tested in a preliminary Analytic Hierarchy Process with *N* = 202 patients and *N* = 36 experts (physicians, nutritionists, diabetes advisors, specialized diabetes nurses). The AHP included attributes on health-related quality of life as well as clinical aspects of diabetes care (outcomes, adverse events as well as co-morbidities).

### Attributes and levels

**Fig. 1:**
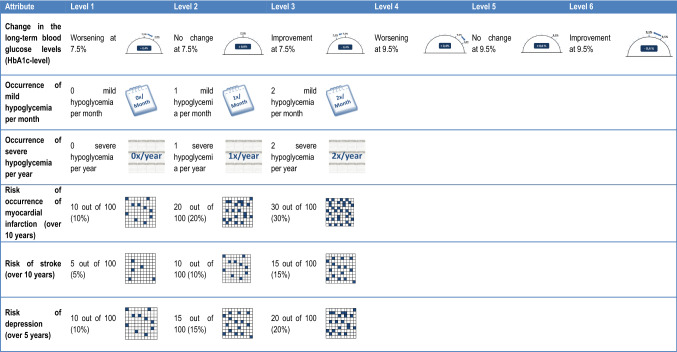
Final decision model of the discrete choice experiments

Following the identification of the most important aspects of the treatment for type II diabetes mellitus and their possible manifestations through the literature research as well as the AHP, the DCE questionnaire was designed. Finally, the final decision model included six attributes (Fig. 1). The “change in the long-term blood glucose levels” has been described as a so-called “compound attribute” by two dimensions: the initial level and the change in percent.

These attributes were used as a basis for determining the importance of the features in the treatment of type II diabetes mellitus with personalized diabetes management and transferred into a standardized online questionnaire. Within the survey, all attributes included amateur-language descriptions. The description of the characteristics has been supplemented by a graphical representation. The objective was to simplify the decision by optimizing the presentation of the information.

### Data collection: experimental design, election scenarios and survey

#### Study population

The empirical assessment of the patient’s preferences was concentrated on insulin-treated patients with the indication diabetes mellitus type II. Patients’ eligibility was linked to the following access criteria: age (18 years or older), diagnosis (diabetes mellitus type II), therapy (regular insulin administration), language skills (sufficient knowledge of German) and given informed consent.

The participants were recruited in cooperation with an external market research company. Data collection took place between 07.06.2017 and 17.06.2017.

According to the formula of Orme [36], at least *N* = 125 subjects were necessary to estimate the main effects (with 12 choice tasks, 2 alternatives and a maximum of 6 variants). Since the current discussion indicates this value as the absolute lowest limit [37], a sample size of *N* = 400 volunteers was applied to guarantee a statistically robust estimate and to carry out the analysis of subgroups.

#### Legal requirements

This study was an anonymous data collection. The Institutional Ethics committee of Hochschule Neubrandenburg has reviewed and approved the interviews and the survey. All subjects submitted an informed consent prior to the first question of the survey and thus agreed to participate in the research project. All subjects were informed about the research project. The participating subjects were free to answer the questions addressed to them or to end the questionnaire at any time during the survey.

#### Experimental design

The software Ngene 1.2.0. [38] was used for optimal experimental design (3 × 5 + 6 × 1 design). The chosen d-efficient design with zero prior estimates included 144 choices divided into 12 blocks, each with 12 choice tasks. The allocation of the participants to the individual blocks was randomized.

Based on the experimental design, two alternatives were displayed (binary choice sets). No status quo alternative was considered. Furthermore, there was no possibility for respondents to choose none of the presented alternative alternatives (None option). Respondents had to choose an alternative (forced choice).

#### Data analysis

The socio-demographic data collected in the first part of the questionnaire were analyzed using descriptive analyzes. The relative importance of each treatment attribute and level was estimated by means of multivariate methods (e.g. random parameter logit model, conditional logit model, latent class model) [39]. SPSS and STATA were used as analysis packages. In all analyzes, *p* < 0.05 (double-sided) was assumed as statistically significant. The evaluation of the preference coefficients took place with consideration of the 95% confidence interval. To explore the determining personality characteristics for class membership in the latent class analysis, cross tables and regression models (binary logistic and multinomial logistic regressions) were used to describe the class distribution by means of the significant structural parameters.

## Results: what do patients prefer?

### Consistency tests to verify response quality

The total respondent sample included *N* = 402 patients. However, it is important to test the consistency of the responses to assess the reliability and validity of the responses. First, individual responses were examined to determine whether the principle of monotonous preferences was fulfilled. According to this, people always prefer more of every normal good [40, 41]. For this purpose, the first choice scenario was designed as a “dominant pair”. Validity tests found that *N* = 36 respondents failed to choose the better, dominant alternative.

For the “stability test”, the first choice set was repeated at the end of the DCE. Respondents who did not select identical alternatives were also excluded (*N* = 111). The test re-test could indicate that the respondents had a learning effect and therefore answered the repeated question differently. However, a comparison of the models with and without application of the exclusion criteria showed a significant difference regarding model fit criteria (e.g. pseudo *R*^2^, AIC, BIC) and magnitude of the coefficients (which affected the *z*-value and hence the *p*-value). The latter indicated that the excluded respondents were less confident in their choice decisions than the remaining respondents in the final model. Beyond, subsequent analysis showed a greater heterogeneity in the model with all respondents included in contrast to the reduced model. This also indicates that a considerable proportion of the respondents made arbitrary choice decisions.

In a further verification of the response validity, the sample was examined for respondents whose responses indicated that they were apparently not willing to trade between the characteristics. According to Marshall et al. [42] a respondent was classified as unwilling if he chose the same alternative in at least 10 of the 12 choice sets (*N* = 48). Finally, a repeated query on the current treatment of diabetes was used. Since treatment with regular insulin doses was an inclusion criterion for this study, respondents who indicated to be treated only with diet/exercise or only with oral antidiabetics during a repeated interrogation were also excluded (*N* = 12). Finally, *N* = 175 respondents were excluded.

### Socio-demographic data

Socio-demographic data of the final respondent sample of 227 patients is shown in Table 1. Next to that, 56.4% were married, 62.9% were retired and 71.4% indicated their current health status to be excellent, very good or good. The average age was 56.6 years (SD 11.21). Regarding their diabetes type II diagnoses, 0.9% had received their diagnoses less than 6 month ago, 7.0% between 6 and 24 months ago, 15.4% between 2 and 5 years ago and 23.3% between 5 and 10 years ago. 42.3% indicated that the diagnosis was made 10–20 years ago and for another 11.0% this was more than 20 years ago.

**Table 1:** Socio-demographic structure of patient sample

	*N* = 227 (%)
Gender	
Male	150 (66.1)
Female	77 (33.9)
Age	
18–29 years	3 (1.3)
30–39 years	18 (7.9)
40–49 years	32 (14.1)
50–59 years	74 (32.6)
60–69 years	77 (33.9)
70–79 years	22 (9.7)
≥ 80 years	1 (0.4)
Mean (SD)	56.66 (11.21)
Education level	
No degree	31 (13.7)
Junior/middle school certificate	77 (33.9)
Intermediate high school certificate. secondary school certificate)	11 (4.8)
Vocational school/advanced technical certificate	17 (7.5)
Abitur. high school diploma in Germany: university entrance qualification	40 (17.6)
Technical college degree	46 (20.3)
University degree or higher	5 (2.2)
Doctorate	31 (13.7)
Other degree	–
Marital status	
Married	128 (56.4)
Widowed	16 (7.0)
Divorced or separated	35 (15.4)
Single	33 (14.5)
In a relationship. but not married	15 (6.6)
Other	–
Current HbA1c level	
≤ 6%	14 (6.2)
6.1–7.0%	91 (40.1)
7.1–8.0%	85 (37.4)
8.1% 9.0%	21 (9.3)
> 9.0%	9 (4.0)
Not sure	7 (3.1)
Frequency of mild hypoglycemia (last month)	
No mild hypoglycemia	37 (16.3)
1 mild hypoglycemia	54 (23.8)
2 mild hypoglycemia	40 (17.6)
3 mild hypoglycemia	17 (7.5)
4 mild hypoglycemia	5 (2.2)
More than 4 mild hypoglycemia	5 (2.2)
Not sure	6 (2.6)
Never had a mild hypoglycemia	52 (22.9)
Not sure (ever)	11 (4.8)
Frequency of severe hypoglycemia (since diagnosis)	
1 severe hypoglycemia	19 (8.4)
More than 1 severe hypoglycemia	16 (7.0)
Not sure	10 (4.4)
No severe hypoglycemia	182 (80.3)

### Assumption of heterogeneity: random parameter logit

In contrast to a conditional logit, the random parameter logit (also “mixed logit”) takes unobserved heterogeneities between the survey participants into account.

Within the model calculation, effects coding was used (− 1, 0, 1; reference category is the negative sum of the other two) and, therefore, no function was assumed about the attribute levels. With this coding, a coefficient is calculated for each level. The following table and figure illustrate the corresponding values.

**Fig. 2:**
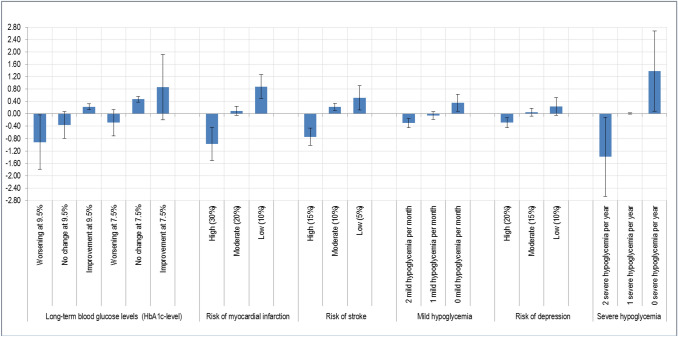
Random parameter model with standard deviations (*N* = 227)

In addition, the random parameter logit allows the assumption of heterogeneous responses within the sample. Since in the model calculation, all attributes were assumed to be random (“random parameter”), the extent of heterogeneous preferences can be derived via the respective standard deviations. The corresponding standard deviations are shown also in Table 2 (below). The graphical representation is shown in Fig. 2.

**Table 2:** Random parameter logit model (coefficients and standard deviations)

Attribute level	Coeff.	SE	*Z*	*p*	CI 95%-low	CI 95%-high	Level diff.
Change in the long-term blood glucose levels (HbA1c level)							1.775
Worsening at 9.5%	− 0.918	0.120	− 7.640	0.000	− 1.153	− 0.682	
No change at 9.5%	− 0.357	0.100	− 3.570	0.000	− 0.552	− 0.161	
Improvement at 9.5%	0.228	0.092	2.470	0.013	0.047	0.409	
Worsening at 7.5%	− 0.285	0.095	− 3.000	0.003	− 0.471	− 0.099	
No change at 7.5%	0.474	0.093	5.100	0.000	0.292	0.656	
Improvement at 7.5%	0.857	0.127	6.740	0.000	0.608	1.106	
Risk of myocardial infarction (over 10 years)							1.854
High (30%)	− 0.975	0.077	− 12.590	0.000	− 1.127	− 0.823	
Moderate (20%)	0.096	0.052	1.840	0.065	− 0.006	0.199	
Low (10%)	0.879	0.075	11.710	0.000	0.732	1.026	
Risk of stroke (over 10 years)							1.255
High (15%)	− 0.741	0.065	− 11.400	0.000	− 0.868	− 0.614	
Moderate (10%)	0.227	0.053	4.250	0.000	0.122	0.332	
Low (5%)	0.514	0.062	8.260	0.000	0.392	0.636	
Occurrence of mild hypoglycemia per month							0.659
2 mild hypoglycemia per month	− 0.301	0.057	− 5.290	0.000	− 0.412	− 0.189	
1 mild hypoglycemia per month	− 0.057	0.052	− 1.090	0.274	− 0.158	0.045	
0 mild hypoglycemia per month	0.358	0.060	5.960	0.000	0.240	0.475	
Risk of depression (over 5 years)							0.523
High (20%)	− 0.287	0.057	− 5.040	0.000	− 0.398	− 0.175	
Moderate (15%)	0.051	0.052	0.990	0.324	− 0.050	0.152	
Low (10%)	0.236	0.059	4.010	0.000	0.121	0.351	
Occurrence of severe hypoglycemia per year							2.765
2 severe hypoglycemia per year	− 1.387	0.106	− 13.090	0.000	− 1.594	− 1.179	
1 severe hypoglycemia per year	0.008	0.050	0.160	0.869	− 0.090	0.107	
0 severe hypoglycemia per year	1.378	0.104	13.250	0.000	1.174	1.582	

	SD*	SE	*Z*	*p*	CI 95%-low	CI 95%-high	
Change in the long-term blood glucose levels (HbA1c-level)							
Worsening at 9.5%	0.878	0.136	6.480	0.000	0.612	1.1434	
No change at 9.5%	− 0.433	0.196	− 2.210	0.027	− 0.817	− 0.0493	
Improvement at 9.5%	0.096	0.172	0.560	0.578	− 0.241	0.4330	
Worsening at 7.5%	0.425	0.170	2.510	0.012	0.093	0.7581	
No change at 7.5%	0.092	0.137	0.670	0.503	− 0.176	0.3596	
Improvement at 7.5%	− 1.057	0.423	− 2.500	0.012	− 1.887	− 0.2279	
Risk of myocardial infarction (over 10 years)							
High (30%)	0.534	0.078	6.850	0.000	0.381	0.6871	
Moderate (20%)	− 0.150	0.068	− 2.190	0.028	− 0.284	− 0.0158	
Low (10%)	− 0.384	0.110	− 3.510	0.000	− 0.599	− 0.1694	
Risk of stroke (over 10 years)							
High (15%)	0.284	0.091	3.110	0.002	0.105	0.4634	
Moderate (10%)	0.111	0.086	1.300	0.195	− 0.057	0.2786	
Low (5%)	− 0.395	0.124	− 3.170	0.002	− 0.639	− 0.1511	
Occurrence of mild hypoglycemia per month							
2 mild hypoglycemia per month	0.141	0.092	1.540	0.124	− 0.039	0.3212	
1 mild hypoglycemia per month	0.129	0.084	1.540	0.123	− 0.035	0.2928	
0 mild hypoglycemia per month	− 0.270	0.127	− 2.130	0.033	− 0.519	− 0.0216	
Risk of depression (over 5 years)							
High (20%)	− 0.162	0.069	− 2.340	0.019	− 0.297	− 0.0266	
Moderate (15%)	− 0.121	0.089	− 1.360	0.175	− 0.296	0.0539	
Low (10%)	0.283	0.114	2.480	0.013	0.059	0.5074	
Occurrence of severe hypoglycemia per year							
2 severe hypoglycemia per year	1.277	0.104	12.250	0.000	1.073	1.4809	
1 severe hypoglycemia per year	0.023	0.079	0.290	0.773	− 0.132	0.1775	
0 severe hypoglycemia per year	− 1.299	0.137	− 9.450	0.000	− 1.569	− 1.0300	

*N* = 227. Obs.: 5448; Log Lik: − 1305.14; AIC: 2670.29; BIC: 2868.38

*The sign of the estimated standard deviations is irrelevant: interpret them as being positive

In this case, slight standard deviations in the graph indicate a rather homogeneous response behavior. This can be seen, for example, in “mild hypoglycemias”. Here, there are only very small, mostly non-significant standard deviations in the coefficients. Another picture emerges from “risk of myocardial infarction”. Here, the standard deviations in the coefficients for all levels are significantly different from zero, i.e. the preferences of respondents are very heterogeneous.

In the model calculation of the random parameter model, all attributes were assumed to be a “random parameter”, i.e. the calculated coefficients are an average value, the corresponding standard deviation describes the extent of heterogeneous preferences within the sample.

### Subgroup testing: latent class analysis

#### Differences in the preferences according to structural variables

Since the random parameter logit model showed certain heterogeneities in the sample, an explorative analysis was generated using a latent class model.

**Fig. 3:**
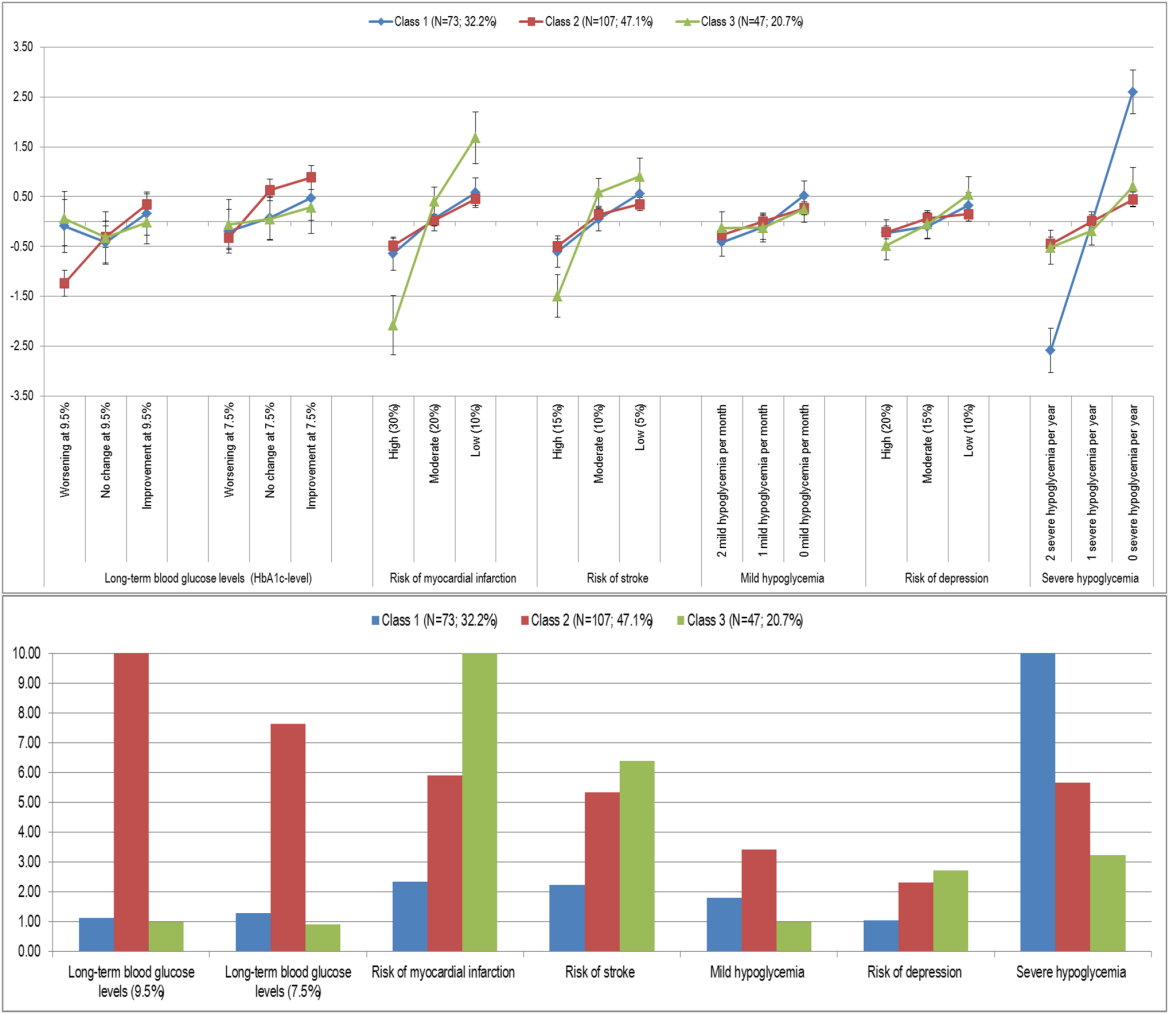
Evaluation of the preference patterns for class 1, 2 and 3 (95% CI) and normalized preference pattern class 1, 2 and 3

To identify possible subgroups, at first a two-class model was developed, followed by a three-class model. A model with four and more classes proved to be meaningfully uninterpretable because of the sample size. The Bayesian Information Criterion (BIC) was used to assess model quality as well as the model best determining existing heterogeneity in response behavior and to provide a probable allocation of the subjects per group. Finally, the three-class model could be identified as suitable. Table 3 shows the results of the preference analysis separated for the three classes (Fig. 3).

**Table 3:** Latent class analysis (3-class-model)

Attribute level	Class 1			Class 2			Class 3		
	Coeff.	SE	*p*	Coeff.	SE	*p*	Coeff.	SE	*p*
Change in the long-term blood glucose levels (HbA1c-level)									
Worsening at 9.5%	− 0.093	0.272	0.732	− 1.236	0.131	0.000	0.057	0.279	0.838
No change at 9.5%	− 0.420	0.220	0.056	− 0.305	0.108	0.005	− 0.318	0.263	0.227
Improvement at 9.5%	0.160	0.222	0.472	0.344	0.107	0.001	− 0.016	0.219	0.940
Worsening at 7.5%	− 0.196	0.223	0.380	− 0.321	0.106	0.003	− 0.059	0.254	0.815
No change at 7.5%	0.077	0.220	0.727	0.633	0.109	0.000	0.052	0.218	0.812
Improvement at 7.5%	0.472	0.228	0.039	0.884	0.121	0.000	0.285	0.269	0.290
Risk of myocardial infarction (over 10 years)									
High (30%)	− 0.645	0.170	0.000	− 0.481	0.075	0.000	− 2.081	0.304	0.000
Moderate (20%)	0.070	0.130	0.591	0.027	0.061	0.664	0.400	0.146	0.006
Low (10%)	0.575	0.151	0.000	0.454	0.071	0.000	1.681	0.264	0.000
Risk of stroke (over 10 years)									
High (15%)	− 0.606	0.162	0.000	− 0.492	0.073	0.000	− 1.494	0.218	0.000
Moderate (10%)	0.049	0.119	0.683	0.143	0.059	0.015	0.587	0.143	0.000
Low (5%)	0.558	0.144	0.000	0.349	0.069	0.000	0.907	0.186	0.000
Occurrence of mild hypoglycemia per month									
2 mild hypoglycemia per month	− 0.413	0.145	0.005	− 0.270	0.073	0.000	− 0.129	0.166	0.439
1 mild hypoglycemia per month	− 0.107	0.128	0.403	0.001	0.063	0.990	− 0.121	0.149	0.416
0 mild hypoglycemia per month	0.520	0.149	0.000	0.270	0.066	0.000	0.250	0.135	0.065
Risk of depression (over 5 years)									
High (20%)	− 0.225	0.132	0.087	− 0.217	0.066	0.001	− 0.484	0.147	0.001
Moderate (15%)	− 0.095	0.131	0.467	0.068	0.061	0.269	− 0.057	0.142	0.691
Low (10%)	0.320	0.131	0.015	0.150	0.071	0.036	0.540	0.186	0.004
Occurrence of severe hypoglycemia per year									
2 severe hypoglycemia per year	− 2.584	0.227	0.000	− 0.451	0.074	0.000	− 0.516	0.175	0.003
1 severe hypoglycemia per year	− 0.014	0.109	0.900	0.009	0.061	0.882	− 0.183	0.146	0.210
0 severe hypoglycemia per year	2.597	0.224	0.000	0.442	0.076	0.000	0.699	0.197	0.000
Patients	*N* = 73 (32.2%)			*N* = 107 (47.1%)			*N* = 47 (20.7%)		
Constant (SE)	− 0.2385 (0.1911)			− 0.7588 (0.2440)			(Reference)

Overall, there were five structural and personality traits which influenced the respective probability of the subjects belonging to one class. As indicators for the group differences, “age” (age groups), the “subjective assessment of one’s own state of health” (based on Likert scale), the “time since diagnosis” (time span), “medical care” (mainly treated by GP or specialist) as well as the “presence of a depression” (diagnosed as co-morbidity) were identified. The three preference patterns were less dependent on the other socio-demographic and therapy-specific factors (Table 4).

**Table 4:** Structural and personality traits per class

Structural and personality traits	Class 1	Class 2	Class 3
Age			
18–39 years		++	
40–69 years	++		−
≥ 70 years			+
Subjective assessment of own health			
Excellent/ very good		++	
Completely dissatisfied	++		−
Time of diagnosis			
≤ 1 year		++	−−
2–5 years	+		−−
> 5 years	−		++
Co-morbidity: depression	+	−−	+
Medical care by			
General Practitioner/family physician	+		
Diabetes specialist practice		+	

−, −−  low number of respondents, +, ++  high number of respondents

#### Preference pattern of class 1

##### Preference pattern

**Fig. 4:**
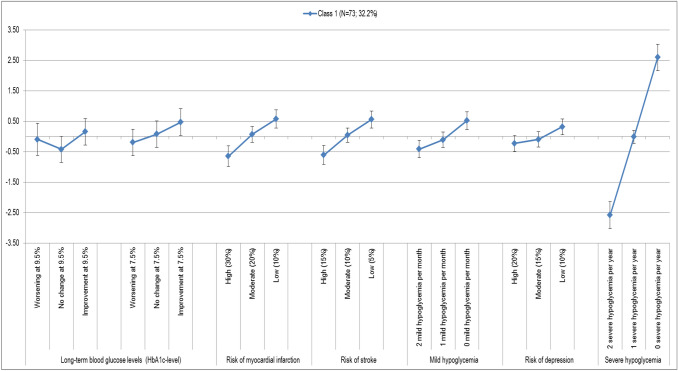
Preference pattern class 1 (95% CI)

The preference pattern of the first class [*N* = 73 (32.2%)] is characterized by a very high rating of severe hypoglycemia, and a clear division of the relevance of the attributes. In this group “occurrence of severe hypoglycemia” is followed by the attributes “risk of heart attack,” “risk of stroke” and the “occurrence of mild hypoglycemia” and the “change in the long-term blood glucose levels.” These attributes are close to each other in their valiancy for the group, the difference of the preference weights is rather small. However, the distance to the first attribute is very high. Finally, the “risk of depression” follows at an even greater distance. This attribute has a weakly significant coefficient only for the lowest level, which would allow the presumption that this side effect has no or very little influence on the decision of this subpopulation (Fig. 4).

##### Evaluation of structural variables

In class 1, the age group of the 40–69 years is most frequently represented (*N* = 64, 88%). In the comparison of the classes, the share of 84% of persons with statutory insurance is the lowest in this class.

Class 1 comprises 67% of all persons of the total sample that were “completely dissatisfied” with their health. In addition, 60% of respondents who reported having experienced more than four mild hypoglycemias last month were grouped in this class.

The participants classified in class 1 also reported an above-average HbA1c level of more than 8.1% (*N* = 11/73, 15%). This is the highest proportion of all classes. The same applies to the fact that 40% of the respondents in class 1 are mainly cared for by a family physician. This is also the highest proportion of all three classes.

#### Preference pattern of class 2

##### Preference pattern

The preference pattern of the second class (*N* = 107; 47.1%) differs significantly from class 1.

**Fig. 5:**
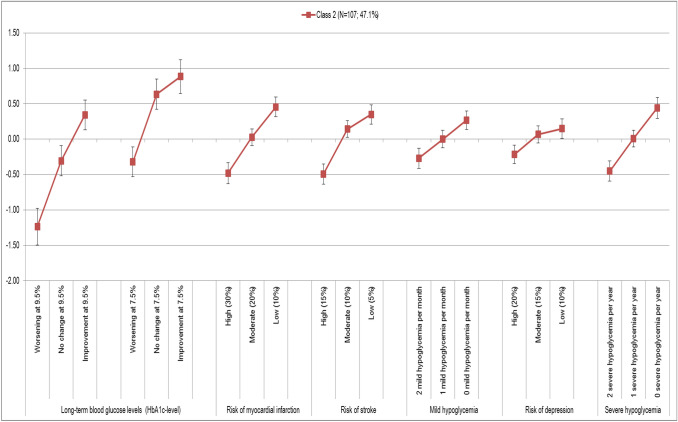
Preference pattern class 2 (95% CI)

Very striking in the evaluation of the preference pattern in class 2 is that the “change of the long-term glucose level” is also the most important attribute in both versions of the compound attribute. A change at an initial value of 9.5% is weighted slightly higher than changes at 7.5% (Fig. 5).

This is identical to the overall evaluation in the random parameter logit model. The placement of the “risk for myocardial infarction” in the first three places is identical to class 1. The “occurrence of severe hypoglycemia” follows closely. Compared to class 1, the “risk of stroke” is ranked fifth. However, it is noticeable in the analysis of class 2 that all attributes in ranks 3–6 are in total denser. The “occurrence of mild hypoglycemia” occupies the penultimate position. As in class 1, “risk of depression” ranks in the last place.

##### Evaluation of the structural variables

The persons grouped in the preference pattern of class 2 above-average said to be aged between 18 and 39 years (*N* = 14/21 in the total sample, 67%). Regarding the self-reported state of health, it is noticeable that 100% of all patients in the total sample, who rated their health as “very good” are grouped in class 2 (*N* = 5/5). In class 2, there are also above-average numbers of patients with a current HbA1c ≤ 6.0% as well as persons who have never experienced a severe hypoglycemia (*N* = 90/181 in the total sample, 50%). This accounts for 84% within this class (*N* = 90/107) and represents the highest percentage within the three classes. Furthermore, only 17% of class 2 participants reported to be diagnosed with depression as a co-morbidity of diabetes. This represents the smallest share of the three classes.

#### Preference pattern of class 3

##### Preference pattern

**Fig. 6:**
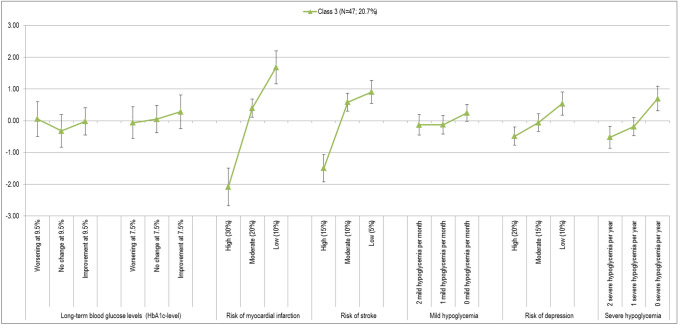
Preference pattern class 3 (95% CI)

A three-part division of the relative importance of the attributes is characteristic of the preference pattern in class 3 (*N* = 47; 20.7%). Unlike the first two groups, the preference pattern in this class is not determined by a characteristic of the glycemic setting, but by co-morbidities of diabetes. The “risk of myocardial infarction” is clearly placed on the first rank. It is followed by another co-morbidity with “risk of stroke”, but with a smaller distance than in the first two classes. The “second” division of attributes is formed by the “occurrence of severe hypoglycemia” and “risk of depression” on the places 3 and 4. To this extent, this group differs significantly from the other two and also the random parameter logit model. It is important to note that the risk of depression is ranked fourth (Fig. 6) and the three least important characteristics of “occurrence of mild hypoglycemia per month” (5th place) and the “change of the long-term glucose level” (place 6 for the initial level of 9.5% and place 7 for changes from 7.5%) have significance values of *p* > 0.1 for all coefficients. This allows the assumption that both attributes did not affect the decision of this subpopulation.

##### Evaluation of structural variables

Individuals who were grouped in the preference pattern of class 3 above-average reported to be male (*N* = 35/47, 75%). In addition, the largest share of pensioners (*N* = 29/47, 61%) of all three groups is in class 3. As a result, respondents in class 3 above-average reported the age group of ≥ 70 years (15%, the highest proportion of all classes). The proportion of participants who have already experienced mild hypoglycemia is also highest in this class (*N* = 40/47, 85%).

In addition, the participants in this class are very experienced with their diabetes. 85% reported having been diagnosed more than 5 years ago. This is the highest proportion of all three classes.

## Discussion

### Descriptive results

The study sample shows a 60/40 distribution of gender in the overall sample for the benefit of the male respondents. This corresponds roughly to the current prevalence and incidence rate in Germany, which also describes a slight accumulation of type II diabetes in the male population [43].

With regard to the level of education, the sample shows an approximate normal distribution in the education level, with slight shifts in favor of the university degree. Identical to other studies, most included subjects were married.

It is striking that the study sample is very experienced with diabetes. 42.3% were diagnosed between 10 and 20 years ago, another 11.0% even more than 20 years ago.

The present study shows a comparable number of diabetic patients treated with insulin therapy compared to other European preference studies in diabetes type II. While the number of subjects in Aristides et al. was *N* = 290 [44], the current study recruited a total of *N* = 227 diabetic patients. Guimarães et al. recruited 274 patients, but not exclusively those with type II [45, 46]. Mühlbacher et al. interviewed *N* = 626 patients with type II diabetes, but the focus here was on therapy with oral antidiabetics [47].

### Rankings and the importance of patient-relevant endpoints

In the conditional logit model as well as the random parameter logit model, all six included attributes had a significance level of *p* ≤ 0.01 (99%) for at least two levels. From this, it can be concluded that the decision-making model included patient-relevant properties. The results of the DCE show that efficacy dominates the side effects in importance. It is noticeable that the “occurrence of severe hypoglycemia” was rated markedly higher, than the “change in the long-term blood glucose levels” or the secondary diseases in almost all analyses. In addition, the Latent Class analysis provides clear indications that there are strong differences between different patient groups and that the overall picture of the random parameter logit model must be considered more differently.

#### Occurrence of severe hypoglycemia

Patients know about the essential meaning of the attribute. This is reflected in the DCE. In the random parameter model, the “change of the long-term glucose levels (HbA1c value)” occupies the first place by far. This is also in line with the results of the AHP. However, it must be mentioned here that the “occurrence of severe hypoglycemia” was placed to the first rank only by the experts. In patients, the characteristic was of lesser importance.

#### Risk of myocardial infarction (over 10 years)

The risk of a myocardial infarction is significantly increased in diabetics compared to non-diabetics. Over a period of 10 years, the incidence of heart disease in healthy people is around 3%, while this rate is between 15 and 20% for diabetics [48]. The “risk of myocardial infarction (over 10 years)” was rated second highest in the overall analysis of the random parameter logit model following the occurrence of severe hypoglycemias. A possible explanation for this is that the affected persons are aware of the correlations between hypoglycemia and the increased risk of myocardial infarction. It has been shown in studies that the first cardiovascular event occurs only an average of 1.5 years after the first onset of hypoglycemia [49].

Patients thus seem to combine a limitation of their performance and a reduction in their quality of life. Nevertheless, the experience with cardiovascular disease seems to be relatively small in the included sample. Only 7.5% of respondents stated that they had already had a heart attack in the past.

#### Risk of stroke (over 10 years)

Like the risk of myocardial infarction, risk of stroke is significantly increased in patients with diabetes mellitus type II compared to the healthy population [48]. This is obviously also registered by the patients and correspondingly taken into account in the evaluation of therapeutic alternatives. Asked about the average 10 years-risk for a patient with diabetes to suffer from a stroke, the majority of patients (75.7%) responded “10–15%” or “even more than 15%”.

Hence, the “risk of stroke (over 10 years)” (taking into account the level difference between the best and the worst level) ranks third in the random parameter logit model.

#### Change in the long-term blood glucose levels

The “change of the long-term blood glucose levels” is weighted fourth and fifth in the random parameter logit model (considering the difference between the best and the worst). The change in the HbA1c value is thus of great importance and a change at an initial value of 9.5% is weighted slightly higher than changes at 7.5%.

The high importance of the HbA1c value has been demonstrated in several studies [50, 51]. The first rank in all models of the present study underlines this high impact and is, therefore, in agreement with the currently available literature on other preference studies. Analogously to the study of Bøgelund et al. [52] and Hauber et al. [53], the attribute “long-term blood glucose level” could be determined as a very important patient-relevant property. Similar results are also found in the study by Mühlbacher/Bethge. Here the “change of the long-term blood glucose” was the second most important criterion in the (hypothetical) therapy decision [47]. The correct setting of the long-term sugar value and the associated short-term and especially long-term prevention of secondary diseases is the ultimate goal of any diabetic therapy [54]. Thus, patient perspective and endpoint relevance are consistent with clinical studies.

#### Occurrence of mild hypoglycemia

The occurrence of mild hypoglycemias per month is placed in sixth place. Hence, the results in this study seem to differ from other preference studies in the evaluation of treatment options in the treatment of type II diabetes. The study by Gelhorn et al. showed a possible hypoglycemia in the first place followed by the adjustment of the HbA1c value [55]. The study by Aristides et al. was able to demonstrate the high significance of the possible hypoglycemia from the patient's perspective. In their preference study in insulin-treated patients the occurrence of hypoglycemia was most important for the patients [44]. However, it must be borne in mind that these preference studies did not distinguish between the occurrence of mild and severe hypoglycemia. This distinction and the consideration of both forms is a special feature of the present study. Gelhorn et al. as well as Aristides et al. related only to “possible hypoglycemias”. If this fact is considered, the results are concordant, because the current study also shows that “hypoglycemias” are the first place and could be identified as crucial patient-relevant characteristic.

Many of the patients surveyed know mild hypoglycemias. When asked whether they had ever experienced mild hypoglycemia, a total of 72.2% of respondents answered “yes”. However, a mild hypoglycemia was only fifth in overall evaluation. It can be assumed that due to the familiarity with this effect and its less severe symptoms/consequences it is of less importance for the patients.

#### Risk of depression (over 5 years)

Along with diabetes, the risk for further complications, such as depression or dementia, increases [17, 56]. Diabetes doubles the risk of depression compared to a healthy person. However, the “risk of depression” is of little or no relevance to diabetic patients facing a decision about a care program. This is shown both in the random parameter logit, as well as in the latent class analysis and is expressed by the relatively low coefficients and the lack of significance in some levels. Thus, this attribute occupies only the last place (taking into account the difference between the best and the worst.) In response to the question of whether a depression/depressive episode has been diagnosed following diabetes, 22.5% of respondents answered “yes”, 67.0% answered in the negative, and 10.6% were “not sure”.

### Heterogeneities: latent class analysis

Based on the model quality criteria, a three-class model was chosen. The three groups of participants show clearly different weights of the therapeutic properties. The number of participants is not fully equally distributed to the three classes: as expected, one group of respondents considers changing the long-term blood glucose (HbA1c value) as the main goal. Another group is solely interested in the short-term effectiveness of diabetes management and thus the reduction in the number of severe hypoglycemias. Surprisingly, another group assessed the risk of cardiovascular problems, such as heart attacks and strokes, the highest.

#### Class 1: Focus on the occurrence of severe hypoglycemia

The preference pattern of class 1 (*N* = 73) is determined by a focus on “occurrence of severe hypoglycemias per year” (LD 5.181, rank 1). The risk of myocardial infarction (over 10 years) (LD 1.219, rank 2) and the risk of stroke (over 10 years) (LD 1164, rank 3) follow at a very great distance. Respondents clearly rate higher numbers of severe hypoglycemia negatively. The focus on the reduction of the frequencies of severe hypoglycemia dominates all other characteristics of care.

Striking in this class is a very clear “threefold” of the attributes. The occurrence of severe hypoglycemias is followed by four attributes (“risk of myocardial infarction”, “risk of stroke”, “occurrence of mild hypoglycemia”, “change of the long-term blood glucose level”) that are closely related in terms of their value for the group. This is illustrated by the small difference in the preference coefficients of these four features. Finally, the “risk of depression” follows at an even greater distance. For this attribute, a weakly significant coefficient can only be calculated for the lowest level, which allows the presumption that this side effect has little or no influence on the decision of this subpopulation.

Class 1 clearly highlights the short-term and immediate success of diabetes management (“occurrence of severe hypoglycemia”). There is a nearly linear course in which the absence of severe events (0× per year) is rated markedly higher than “1 severe hypoglycemia per year”. These characteristics, in turn, clearly dominates the worst level “2× per year”.

This preference pattern is also reflected in the evaluation of the structural variables. It is striking that the class combines the largest proportion of patients who have already experienced severe hypoglycemia (53% of all respondents). This can serve as an explanation approach for the high value of the attribute.

In addition, 67% of all persons in the overall sample who said they were “completely dissatisfied” with their health have high probabilities of belonging to class 1. This fact might also explain why the short-term and potentially directly life-threatening (accompanying) symptoms of diabetes—severe hypoglycemia, myocardial infarction and stroke—are among the top places. Patients in this group are concerned about their health and want to avoid these serious effects.

Furthermore, 54% of all respondents who prefer the doctor to make the final decision on treatment are found in class 1. In addition, 40% of the respondents in class 1 are mainly cared for by the family physician. This is also the highest proportion of all three classes.

In this respect, it can be assumed that the management of diabetes and the doctor-patient communication in this group have a clear influence on the patient's therapeutic preferences. Patients in class 1 seem to follow the recommendations of their (family) doctors on their treatment or everyday life.

#### Class 2: Focus on changing the long-term blood glucose

The objective ranking in class 2 (*N* = 107) is clearly deviating from class 1. Like the first class, a triple division is also recognizable, but with smaller distances in importance. In the second class the “change of the long-term blood glucose levels” is most important (LD 2120). This is particularly striking, since this characteristic is placed first only in this class. In the other two classes, the “change of the long-term blood glucose levels” occupies the fifth place. The ranking of the “risk for myocardial infarction” (LD 0.935) in the second place is identical to class 1, but the difference to the most important attribute is significantly lower than in the first class. With “occurrence of severe hypoglycemias per year” (LD 0.893) and “risk of stroke” (LD 0.841) on the places 3 and 4, it is followed by attributes, which are relatively similar in their relative importance to “risk of myocardial infarction”. These three attributes appear to be valued by the patients in combination.

Class 2 clearly emphasizes the long-term and lasting adjustment of the blood glucose in the context of diabetes management. Only in this class a nearly linear course over all levels of the attribute can be seen. For both hypothetical “initial values” of 7.5 and 9.5% an improvement is valued significantly higher than a constant value. Both clearly dominate the worst level of “deterioration”. It is also noticeable that the changes at an initial value of 7.5% each achieve higher coefficients and consequently were seen as more relevant by the patients in this subgroup.

This preference pattern is also reflected in the evaluation of the structural variables. It is noteworthy that the majority of respondents with a current HbA1c ≤6.0% and 49% of all respondents in the range of 6.1–7.0% were grouped in class 2 (based on the individual probability of class membership). In addition, patients in this class are relatively newly diagnosed with diabetes.

For this class, the expert care and the structured management of their diabetes seem particularly interesting. This is reflected in the fact that most of respondents who are enrolled in a care program, such as DMP, are grouped in class 2. In addition, patients in this class prefer treatment in diabetes centers or specialist practices.

#### Class 3: Focus on cardiovascular events

The third group shows a different picture. For class 3 (*N* = 47) the focus is on avoiding cardiovascular events. Diabetes is a risk to the heart and blood vessels and can lead to congenital damage such as circulatory disorders, increased strokes and heart attacks. This seems particularly relevant to the patient in this class. Consequently, the “risk of heart attack (over 10 years)” (LD 3762) is the most important attribute that dominates all other characteristics. In the second place, a second cardiovascular event follows—“risk of stroke (over 10 years)” (LD 2401). Patients in class 3 focus solely on the risk of these secondary diseases, regardless of the frequency of hypoglycemia or adjustment of the long-term glucose level.

In this class, the “risk of depression (over 5 years)” is ranked fourth and hence ranked highest in the comparison of the three classes. This supports the assumption that the patients in this group focus exclusively on long-term follow-up and secondary diseases when choosing a care program.

Consequently, in this class, the “change of the long-term glucose level” and the “occurrence of mild hypoglycemia per month” fall back on the penultimate and last position. These two attributes do not produce any significant coefficients for any of the levels and are thus irrelevant for this patient group.

82% in class 3 reported suffering from hypertension. This is the highest proportion of all classes. Since hypertension is another risk factor for cardiovascular events, a further explanation for the shown preference pattern could be included herein. In addition, the participants in this class are very experienced with their diabetes. 85% in the class reported having received the diagnosis more than 5 years ago. This is also the highest proportion of all three classes. Finally, above average, they stated to be male. It can be concluded that these experienced, elderly patients are very sensitive to long-term consequences of their diabetes with regard to cardiovascular events, regardless of the short-term effectiveness of a diabetes management.

### Limitations

Although the use of the DCE method in the field of diabetes therapy could be demonstrated, some limitations are to be taken into account in the analysis, interpretation and generalization of the data.

Respondents were recruited by means of an external service provider. This may have influenced the study population regarding individual parameters (selection bias) and cannot be verified in its entirety.

The use of the “forced choice” integrated into the DCE forced participants to choose one of the presented therapy alternatives. This does not necessarily reflect reality and does not mean that the patients would also choose the alternatives shown in reality. A non-forced choice is recommended to forecast willingness to pay or demand. Because this was not the objective of this study, this limitation appears reasonable. The advantage of the chosen variant lies in the data quality.

Attributes in a DCE must be formulated in such a way that respondents clearly and concisely understand the meaning of the attribute. Especially risk attributes should be explained thoroughly in the course of the DCE to avoid mistakes based on difficulties in understanding probabilities and the concept of risk. Most recent studies apply a combination of quantitative description and visual support to present risk attributes. Regarding the number of risk attributes, some studies used the same number of risk and benefits attributes (e.g. [57]), other use more risk than benefit attributes (e.g. [58]) or less risk attributes (e.g. [59]). The use of risk attributes in DCEs is repeatedly highlighted as an area for further research. Basically, the number of risk attributes is discussed less critically than the way in which a risk attribute is to be communicated and presented/framed to respondents. Special attention is needed towards attributes involving a description of risk as risks are often perceived as difficult to interpret. The number of risk attributes within the recommended number of attributes in general in a DCE does not seem to influence the overall result. More important seems to be how respondents perceive and evaluate the risks presented. Since there is no guideline on how to present risk attributes, further research is needed on how to deal with or overcome framing effects [60, 61].

For the experiment, a d-efficient design with prior estimates was used. D-optimal designs attempt to maximize attribute level differences and might lead to lexicographic choice behavior. However, as D-optimal designs focus on the attribute level differences no prior estimates are used for constructing these designs.

The mixed logit model outperformed the conditional logit model in terms of model fit criteria and magnitude of coefficients. All coefficients in the mixed logit model were assumed to be normally distributed. Other distributions were not assumed to be applicable, e.g. uniform. This remains a limitation in the analysis.

In the latent class analysis, the parameter estimates for the middle level of “risk of depression” and “occurrence of severe hypoglycaemia” are not significant. Respondents seem to only value the extreme levels of these attributes. This finding was not yet analyzed in more detail, but it could also be a behavioral issue. Respondents might have clear preferences for the attributes, but behaviorally regard these attributes as a binary outcome. Further analyses will be run on this.

As other studies have shown, preferences can depend on cultural background, expectations and attitudes of the study sample, and the current health system as a context factor [62]. When interpreting and generalizing these results, it should be taken into account that the study was based on a German population and conducted in mid-2017.

Finally, preferences might be influenced by different circumstances. Hence, the given information on attributes and levels, the patient’s background of experience, as well as cognitive abilities are decisive [63, 64]. Preferences can thus vary depending on the decision context.

## Conclusion

Diabetes as a chronic disease places high demands on the compliance and adherence of patients. The success of the therapy is determined by various factors such as adherence to the recommended blood glucose monitoring or the documentation of blood glucose profiles. The use of personalized approaches to diabetes management is discussed on an international level and the success has been demonstrated in the first studies. Studies show that personalized diabetes management can increase the effectiveness and efficacy of therapy and improve treatment outcomes [19–22].

Decisions on diabetes therapy and/or management are very complex and require trade-offs between possible outcomes and simultaneous risks and secondary diseases. The present data show that patients have the highest weight on the “occurrence of severe hypoglycemias per year”. On the second place is “change of the long-term blood glucose level (HbA1c level)”, followed by “risk of myocardial infarction (over 10 years)” (considering the 95% confidence interval). Thus, the reduction of the metabolic derivate as well as the optimal adjustment of the HbA1c level within the framework of a possible choice of a management approach has the decisive importance for the diabetic patients.

Even if it appears surprising that the “occurrence of severe hypoglycemia” is weighted higher than the “change in long-term blood glucose level” from patients’ perspective, this can be supported by current studies. In the sample, more than 75% of respondents stated that they were older than 50 years. According to recent studies from the USA, a “normal” setting of the blood glucose can already mean an over-therapy, especially in elderly and comorbid diabetic patients. For them, the risks outweigh the desired benefits of drug treatment [65].

Considering the limitations, the results are also valuable for the doctor–patient relationship and the diabetes management. The DCE provides a practical approach that can help improve communication between patients and care providers. This approach has the potential to support clinical and allocative decision-making and to improve the quality of interpretation of clinical data in the long term. Diabetes management and therapies can be designed more patient-oriented based on the findings obtained. In addition, more effective and efficient care can be achieved and patient benefit increased [66].
